# The distinct functional brain network and its association with psychotic symptom severity in men with methamphetamine-associated psychosis

**DOI:** 10.1186/s12888-024-06112-4

**Published:** 2024-10-10

**Authors:** Zhen-An Hwang, Ai-Ling Hsu, Chia-Wei Li, Changwei W. Wu, Chun-Hsin Chen, Wing P. Chan, Ming-Chyi Huang

**Affiliations:** 1 Department of Radiology, Wan Fang Hospital, Taipei Medical University, Taipei, 116 Taiwan; 2 Department of Radiology, School of Medicine, College of Medicine, Taipei Medical University, Taipei, Taiwan; 3 Department of Artificial Intelligence, Chang Gung University, Taoyuan, Taiwan; 4 Department of Psychiatry, Chang Gung Memorial Hospital at Linkou, Taoyuan, Taiwan; 5 Graduate Institute of Mind, Brain and Consciousness (GIMBC), Taipei Medical University, Taipei, Taiwan; 6 Department of Psychiatry, Wan Fang Hospital, Taipei Medical University, Taipei, Taiwan; 7 Department of Psychiatry, School of Medicine, College of Medicine, Taipei Medical University, Taipei, Taiwan; 8 Psychiatric Research Center, Wan Fang Hospital, Taipei Medical University, Taipei, Taiwan; 9 Department of Addiction Sciences, Taipei City Psychiatric Center, Taipei City Hospital, Taipei, Taiwan

**Keywords:** Brain, Functional magnetic resonance imaging (fMRI), Methamphetamine, Neurotransmitter, Psychosis

## Abstract

**Background:**

Individuals using methamphetamine (METH) may experience psychosis, which usually requires aggressive treatment. Studies of the neural correlates of METH-associated psychosis (MAP) have focused predominantly on the default mode network (DMN) and cognitive control networks. We hypothesize that METH use alters global functional connections in resting-state brain networks and that certain cross-network connections could be associated with psychosis.

**Methods:**

We recruited 24 healthy controls (CRL) and 54 men with METH use disorder (MUD) who were then divided into 25 without psychosis (MNP) and 29 with MAP. Psychotic symptom severity was assessed using the Positive and Negative Syndrome Scale (PANSS), evaluating (1) large-scale alterations in regional-wise resting-state functional connectivity (rsFC) across 11 brain networks and (2) associations between rsFC and psychotic symptom severity.

**Results:**

The MUD group exhibited greater rsFC between the salience network (SN)-DMN, and subcortical network (SCN)-DMN compared to the CRL group. The MAP group exhibited decreased rsFC in the sensory/somatomotor network (SMN)-dorsal attention network (DAN), SMN-ventral attention network (VAN), SMN-SN, and SMN-auditory network (AN), whereas the MNP group exhibited increased rsFC in the SMN-DMN and the frontoparietal network (FPN)-DMN compared to CRL. Additionally, the MAP group exhibited decreased rsFC strength between the SMN-DMN, SMN-AN, SMN-FPN, and DMN-VAN compared to the MNP group. Furthermore, across the entire MUD group, the PANSS-Positive subscale was negatively correlated with the DMN-FPN and FPN-SMN, while the PANSS-Negative subscale was negatively correlated with the DMN-AN and SMN-SMN.

**Conclusion:**

MUD is associated with altered global functional connectivity. In addition, the MAP group exhibits a different brain functional network compared to the MNP group.

**Supplementary Information:**

The online version contains supplementary material available at 10.1186/s12888-024-06112-4.

## Background

Methamphetamine (METH) is a highly addictive psychostimulant that principally affects brain neurotransmitter systems, particularly dopamine, and produces an increase in alertness, energy, and euphoria [1]. With the increasing prevalence of METH exposure globally, converging evidence indicates that repeated METH exposure causes a variety of deleterious physical and psychological consequences [2–4]. Among them, METH-associated psychosis (MAP) has been of particular concern because of its association with an increased risk of suicide and violence, poorer cognitive function, and increased health service utilization [5, 6]. Compared to the general population, individuals who use METH are 11 times more likely to develop psychotic symptoms [7]. Additionally, most experience “positive” psychotic symptoms during intoxication, typically lasting no more than one week [8]. Treating MAP is particularly important because the affected population is at a very high risk of experiencing symptom exacerbation, and it is associated with a poorer prognosis [9]. Understanding how the neurobiological features of MAP are distinct and different from METH without psychosis (MNP) will ultimately optimize therapeutic strategies and reduce the risks of unfavorable outcomes.

Current evidence suggests that the difference between health and disease, or between the presence and absence of nominally psychiatric symptoms, is not defined by sharp or discontinuous neurobiological boundaries [10]. In light of this, some reports indicate that psychotic symptoms are a consequence of disordered connectivity within and between distributed brain networks [11, 12]. In the context of METH use, both animal and human studies have shown that differences in METH-related neuropsychiatric functions could be reflected in variability across the collective set of neural systems [2, 13]. Studies using task-based functional magnetic resonance imaging (fMRI) have demonstrated the impact of METH on various brain networks that underlie multiple neurocognitive domains such as reward learning [14], decision making [15], and adaptive cognitive control [16]. Although there have been an increasing number of studies linking brain network functioning with neuropsychiatric disturbances following METH administration, we are far from understanding how brain networks might explain the differences between MAP and MNP.

Beyond focusing on alterations of task-driven brain regions or networks, resting-state functional connectivity (rsFC) MRI technique has been widely used to investigate alterations in large-scale functional organizations of brain networks under neuropsychological conditions when people are not engaged in a specific task [17, 18]. To date, only a few human neuroimaging studies have focused on exploring functional network disruptions between MAP and MNP [19]. For example, using the rsFC MRI technique, Ipser et al. identified abnormal connectivity between the default mode network (DMN) and the cognitive control network (CCN) in these two groups of patients [20]. Furthermore, connectivity between these two networks was found to be inversely associated with the duration of antipsychotic treatment among MAP patients, indicating a relationship between connectivity and psychotic severity [20]. Similarly, a more recent study found that, compared to controls, the connectivity distinctions of MAP involved several networks such as attention, memory, and self-processing; however, a negative correlation between connectivity in the frontal gyrus and psychotic illness was reported [21]. Despite the literature describing regional and cross-network rsFC abnormalities in the population with MAP, a consensus has not yet been reached as to how connectome disturbances associated with psychotic severity are systematically manifested.

Given that individuals with MNP can benefit from substance use treatment, whereas those with MAP could require additional antipsychotic treatment [22], exploring the neuroimaging characteristics associated with MAP could provide insights into the appropriate placement of clinical treatment. In this study, we investigated rsFC alterations using pairwise comparisons between control participants and individuals with METH use disorder (MUD), including both MNP and MAP patients, to unravel the METH-induced psychotic effect from METH exposure based on large-scale brain functional networks. Furthermore, we examined the association between functional connections and the severity of psychotic symptoms. Identifying these altered networks could facilitate future classification approaches for METH users with and without psychoses.

## Methods

All participants were given a full explanation of the study procedure and were asked to provide written informed consent as per the guidelines of the Research Ethics Committee (IRB No. TCHIRB-10902009). Treatment-seeking individuals who used METH were recruited from the Department of Addiction Sciences at the Taipei City Psychiatric Centre. Neuroimaging was performed at the Department of Radiology at Wang Fang Hospital in Taipei.

### Participants

#### MUD group

The inclusion criteria were: (1) Men aged between 20 and 45 years; (2) fulfilling the criteria of the Diagnostic and Statistical Manual of Mental Disorders, Fifth Edition (DSM-5) for MUD as verified by two board-certified psychiatrists (coauthors M.C.H. and C.H.C.); (3) positive urine toxicology test for METH when treatment was administered; and (4) no MRI incompatibilities or known claustrophobia. Exclusion criteria were: (1) history of any other substance use disorder except for nicotine; (2) history of schizophrenia, bipolar disorder, or major depressive disorder; (3) history of major systemic illness such as hypertension, cardiovascular disease, diabetes mellitus, or thyroid, renal, or liver disease; (4) history of epileptic seizures, head injury, loss of consciousness, or neurological disorders; and (5) use of benzodiazepines one week prior to MRI examination. We have implemented the Chinese version of Diagnostic Interview of Genetic Studies (DIGS) [23, 24], a semi-structured diagnostic interview for psychiatric disorders, to collect sociodemographic data and METH use variables, and to screen for psychotic symptoms, including delusions and hallucinations. These symptoms were further confirmed by M.C.H. and C.H.C., who followed the participants for a month to verify the presence of METH-induced psychotic symptoms, such as delusions, hallucinations, suspiciousness, and unusual thought content, as suggested previously [7, 8]. Accordingly, those with psychotic symptoms were classified as the MAP subgroup, and those without psychotic symptoms were classified as the MNP subgroup.

#### Healthy control group

Healthy sex-, age-, and education-matched volunteers were recruited from the community through advertisements. Recruitment criteria were: (1) aged between 20 and 45 years; (2) no history of any substance use disorders except nicotine; (3) no history of major psychiatric, systemic, or neurological diseases, as described above; and (4) no MRI incompatibilities or known claustrophobia. In addition, the handedness of the healthy controls and MUD groups was recorded.

#### Psychological assessments

Because METH withdrawal lasts for 2–4 weeks [25], clinical data were evaluated after 3–4 weeks, when the participant was clear of withdrawal symptoms. A trained research assistant conducted face-to-face interviews, employing the Hollingshead Four-Factor Index of socioeconomic status (SES) scale [26–28] and the Chinese version of DIGS [23, 24]. The severity of psychotic symptoms was assessed using the Chinese version of the Positive and Negative Syndrome Scale (PANSS) (M.C.H.) [29]. Depressive and anxiety symptoms were evaluated using the Chinese versions of the 21-item Beck Depression Inventory (BDI) [30] and Beck Anxiety Inventory (BAI) [31].

#### MRI data acquisition

All datasets were acquired using a 3-T scanner (Discovery MR750w; GE Healthcare, Milwaukee, USA) with a 24-channel brain coil at Wan Fang Hospital. The protocol included rs-fMRI scans using a T2* weighted gradient echo-planar imaging sequence (repetition time/echo time, 2500 ms/30 ms; flip angle, 80°; 43 slices at a thickness of 3 mm with no gap; in-plane resolution, 3 × 3 mm^2^; 210 volumes), followed by a three-dimensional T1 weighted scan using a brain volume gradient echo sequence (FSPGR-BRAVO) (repetition time, 8.5 ms; echo time, 3.248 ms; time to inversion, 450 ms; flip angle, 12°; field of view matrix size, 256 × 256; voxel size, 0.94 × 0.94 × 1.2 mm^3^). The total MRI scan time was approximately 40 min. During the resting-state scan, participants were asked to keep their eyes closed, to remain stationary but avoid falling asleep, and not to think about anything in particular during the 6-minute acquisition period of 210 volumes, of which the first ten were discarded.

#### Functional MRI analysis

All resting-state functional MRI (rs-fMRI) data preprocessing and subsequent computation of the whole-brain functional connectome were performed using the open-access IClinfMRI [32] and AFNI software [33], respectively. Data preprocessing involved correcting each rs-fMRI volume for head movement using a rigid-body spatial transformation, applying nonlinear normalization to the standard Montreal Neurological Institute space, and then resampling to 2-mm isotropic voxels. Following normalization, high-motion spikes and third-order trends presented in the rs-fMRI data were removed through volume censoring (*3dDespike*) and detrending (*3dDetrend*) methods to reduce voxel-wise effects of head movement and drifting artifacts. Multiple linear regression was then employed to eliminate the nuisance effects from motion profiles and physiological fluctuations from white matter and cerebrospinal fluid. Subsequently, band-pass filtering of rs-fMRI fluctuations within a range of 0.01–0.08 Hz was applied in order to preserve neural frequencies while reducing motion-induced frequencies, followed by smoothing at 4-mm full-width at half-maximum. To address potential negative influence attributed to head motion during data acquisition, we implemented a 5-stage retrospective strategy as recommended [34]. The first four stages were performed in individual preprocessing, including realignment, volume censoring, nuisance regressor (individual-level generalized linear model, GLM), and band-pass filtering. The last stage involved regressing out the individual mean framewise displacement (FD) during group analysis. After the above preprocessing, the whole-brain functional connectome was then examined, reflecting inter-regional communications within a predefined framework of eleven functional networks [35]: dorsal attention network (DAN), ventral attention network (VAN), subcortical network (SCN), salience network (SN), frontoparietal network (FPN), visual network (VN), default mode network (DMN), auditory network (AN), cingulo-opercular network (CON), sensory/somatomotor network (SMN), and memory networks (MN). The construction of the whole-brain functional connectome was based on a set of region of interests (ROIs) defined with this functional network framework, where each ROI was represented by a sphere with a 5-mm radius. To comprehensively analyze the functionality disruptions in terms of the functional network framework following METH administration, 245 ROIs derived from three sources were integrated. The sources were: (1) 229 ROIs summarized from previous task-evoked fMRI and rs-fMRI studies in the meta-analytic literature [36]; (2) 12 ROIs from an rs-fMRI study that thoroughly modeled the basal ganglia circuitry [37]; and (3) 4 ROIs (2 amygdala and 2 hippocampus) to complement the SCN, sourced from large-scale automated syntheses using “emotion” and “memory” terms, respectively, spanning 1037 and 2744 studies, respectively, in Neurosynth [38]. The detailed coordinates of each ROI are shown in Supplementary Table 1. Correlation coefficients between the preprocessed time series of each ROI pair in the functional network framework were computed using a Pearson correlation, as implemented in the AFNI 3dNetCorr method [39]. For each participant, a 245 × 245 correlation matrix was produced and subsequently transformed into a *z*-transformed matrix using Fisher’s *r-to-z* transformation. Specifically, these connectivity matrices include links for both the intra- and inter-network connections among the 11 large-scale functional networks.

#### Motion analysis of rs-fMRI data

For each participant, the motion profiles of the six motion parameters, encompassing translations and rotation along a three-dimensional coordinate system, were summarized as the mean frame displacement (FD) metric.

### Statistical analysis

To analyze demographic and clinical characteristics, as well as motion profiles of rs-fMRI data, one-way ANOVA and chi-square tests were conducted to examine the main effects using SPSS software (version 22, IBM, Chicago, IL). For data distribution violating the normality assumption, as determined by the Shapiro–Wilk test, the non-parametric Kruskal–Wallis test was used instead. Beyond the main effect, post-hoc pairwise comparisons with Bonferroni corrections were applied to examine significance between groups. The statistical threshold was set at *p* < 0.05, two-tailed.

In this study, two primary statistical analyses of the whole-brain functional connectome were performed using the Network-Based Statistic method [40]. Link-based statistical tests were applied to identify significant changes in pairwise functional matrices within and between the 11 large-scale functional networks. These changes were examined across all possible pairings of the three participant groups: CRL, MNP, and MAP. To mitigate the effects of head movement at the population level, the mean FD for each participant was included as a nuisance regressor. Additional nuisance regressors accounted for potential confounding factors, including age and education, which may interact with METH dependence. Besides, we controlled for depression (BDI) and anxiety (BAI) scores to disentangle the effects of psychosis from those of depression and anxiety states. All nuisance regressors were centered on their mean during statistical testing [41].

In addition to examining group differences among the three groups, the relationship between connectivity and the severity of psychotic symptoms within the MUD group (*n* = 54) was examined using regression analysis. To account for multiple comparisons in both the group comparison and the regression analysis, the significance level was corrected using a false discovery rate, *p* < 0.05, and 5,000 permutations.

## Results

### Sociodemographic, clinical characteristics, and rs-fMRI motion profiles

**Fig. 1:**
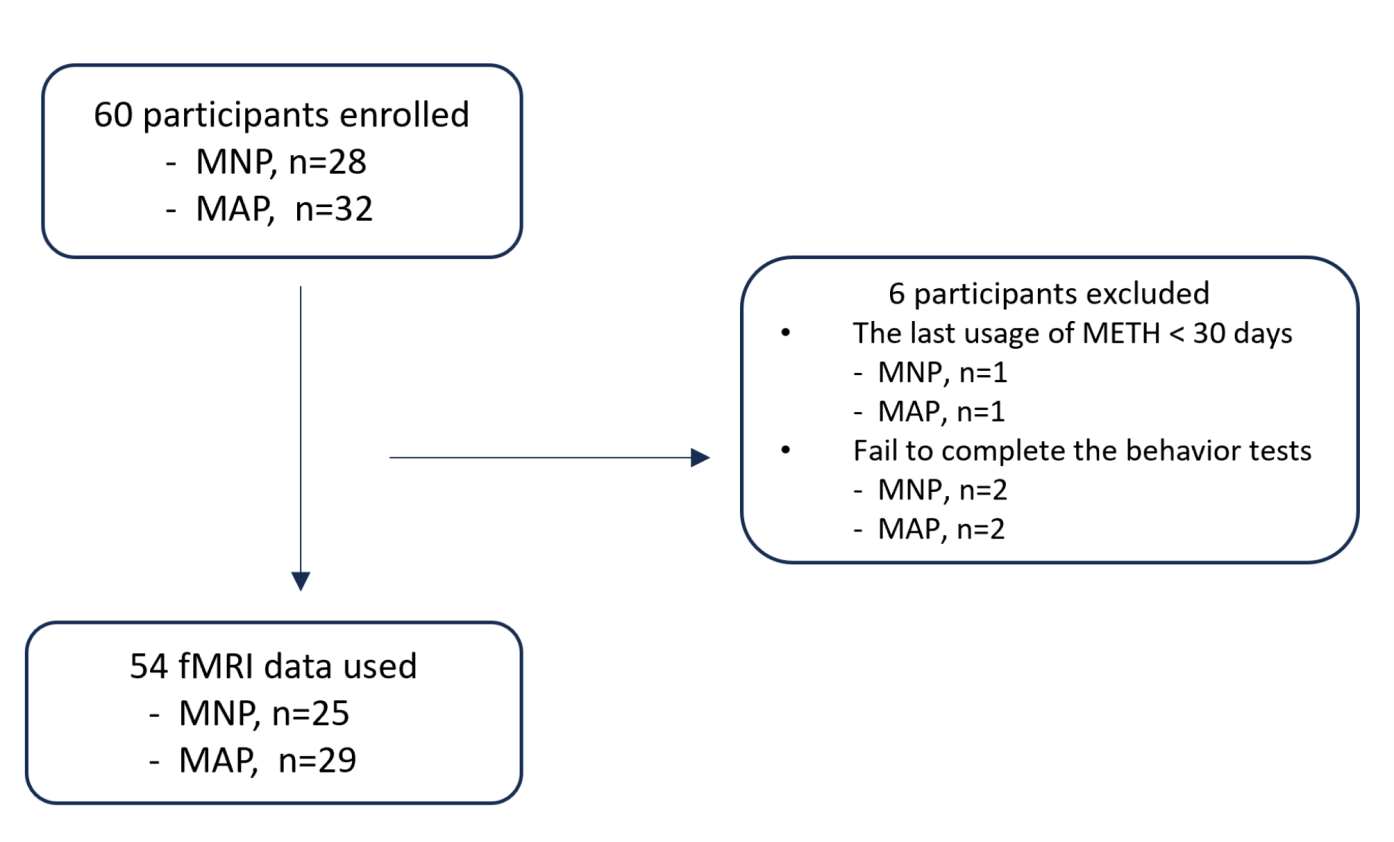
Flow diagram of study participants

Of the 60 men enrolled in the MUD group, 6 were excluded due to recent (within 30 days) exposure to METH (*n* = 2) or incomplete psychiatric assessment results (*n* = 4). The remaining 54 participants were classified into the MAP subgroup (*n* = 25) or the MNP subgroup (*n* = 29) (Fig. 1). Table 1 summarizes the demographic and clinical characteristics of each participant group. No significant differences were observed between the three groups regarding age, years of education, socioeconomic status, or handedness. Furthermore, the MAP and MNP subgroups did not differ in age at initiation of METH use, duration of use, or severity of psychotic symptoms (PANSS-Positive [PANSS-P], PANSS-Negative [PANSS-N], PANSS-General Psychopathology [PANSS-GP], and PANSS-Total [PANSS-T]). In post-hoc analysis, the MAP subgroup exhibited significantly higher scores on the BAI compared to the MNP subgroup (*p* < 0.05 after Bonferroni correction). However, there was no significant difference in the BDI between these two subgroups. Significant differences in both BDI and BAI scores were observed between the CRL and MAP groups (*p* < 0.05 after Bonferroni correction). Additionally, years of smoking and pack years showed significant differences between the CRL group and both the MNP and the MAP subgroups (*p* < 0.05 after Bonferroni correction). Regarding head-motion characteristics during the rs-fMRI scan, no participants exhibited maximum head movement (displacements ≥ 4.0 mm or rotations ≥ 3.0° in any direction). The Kruskal-Wallis H test showed no significant difference in mean FD across the three groups, χ^2^(2) = 1.46, *p* > 0.48.

**Table 1:** Demographic and clinical variables between healthy controls and MUD group

	CRL	MUD patients		Main effect	Post-hoc pairwise comparisons		
		MNP	MAP		CRL vs. MNP	CRL vs. MAP	MNP vs. MAP
Variables	*n* = 24	*n* = 25	*n* = 29				
Age, years old,	32.1 ± 5.1	35.1 ± 8.0	32.7 ± 6.0	0.24^k^			
Education (years)	13.6 ± 2.3	14.0 ± 2.3	14.3 ± 2.7	0.61^k^			
SES (range)	35.1 ± 10.3 (range 13–52)	34.5 ± 12.7 (range 16–61)	29.2 ± 10.3 (range 11–51)	0.11^k^			
Smokers, n (%)	3(12.5%)	13(52%)	18(62%)	**< 0.001** ^**c**^	**0.001** ^#^	**0.521**	0.056
Years of Smoking	2.4 ± 6.7	10.2 ± 12.3	9.5 ± 9.7	**< 0.005** ^**k**^	0.012*	**0.004** ^#^	1.000
Average package of smoking per day	0.08 ± 0.24	0.3 ± 0.36	0.6 ± 1.04	**0.002** ^**k**^	0.032*	0.001^#^	1.000
Pack Years	1.7± 5.5	5.4 ± 7.1	10.4± 18.9	**< 0.001** ^**k**^	0.019*	0.002^#^	1.000
Age of first use of METH, years old	N/A	30.4 ± 9.7	26.1 ± 6.6	0.06 ^m^			
Duration of METH use, years	N/A	3.7 ± 4.5	4.5 ± 4.3	0.10^k^			
Handedness				0.98^c^			
Right	22	23	27				
Left	2	2	2				
**Psychological assessment**							
BDI (0–63)	5.2 ± 6.3	9.3 ± 8.9	14.0 ± 10.9	**< 0.001** ^**k**^	0.247	**0.001** ^#^	0.151
BAI (0–63)	2.8± 4.7	3.4± 5.5	9.7 ± 10.5	**< 0.005** ^**k**^	1.000	**0.005** ^#^	**0.021** ^*^
PANSS Total		37.9 ± 5.2	39.4 ± 5.1	0.20^m^			
PANSS-Positive		8.3 ± 1.6	8.9 ± 2.2	0.36^m^			
PANSS-Negative		8.9 ± 1.7	9.0 ± 2.3	0.62^m^			
PANSS-GP.		20.7± 3.4	21.6 ± 3.0	0.23^m^			

^k^: Kruskal Wallis test, ^m^: Mann–Whitney U test, ^c^: Chi–square test

*: *p* < 0.05 after Bonferroni correction; #: *p* < 0.01 after Bonferroni correction. The p-values in bold represent those *p* < 0.05

Abbreviations: CRL: healthy controls; MUD: methamphetamine use disorder; MNP: methamphetamine users with no psychosis; MAP: methamphetamine-associated psychosis; N/A: not applicable; SES: Hollingshead Four-Factor Index of SocioEconomic status scale; BDI: Beck Depression Inventory; BAI: Beck Anxiety Inventory; PANSS: Positive and Negative Syndrome Scale; GP: General Psychopathology

### Group differences in inter-network and intra-network functional connectivity

**Fig. 2:**
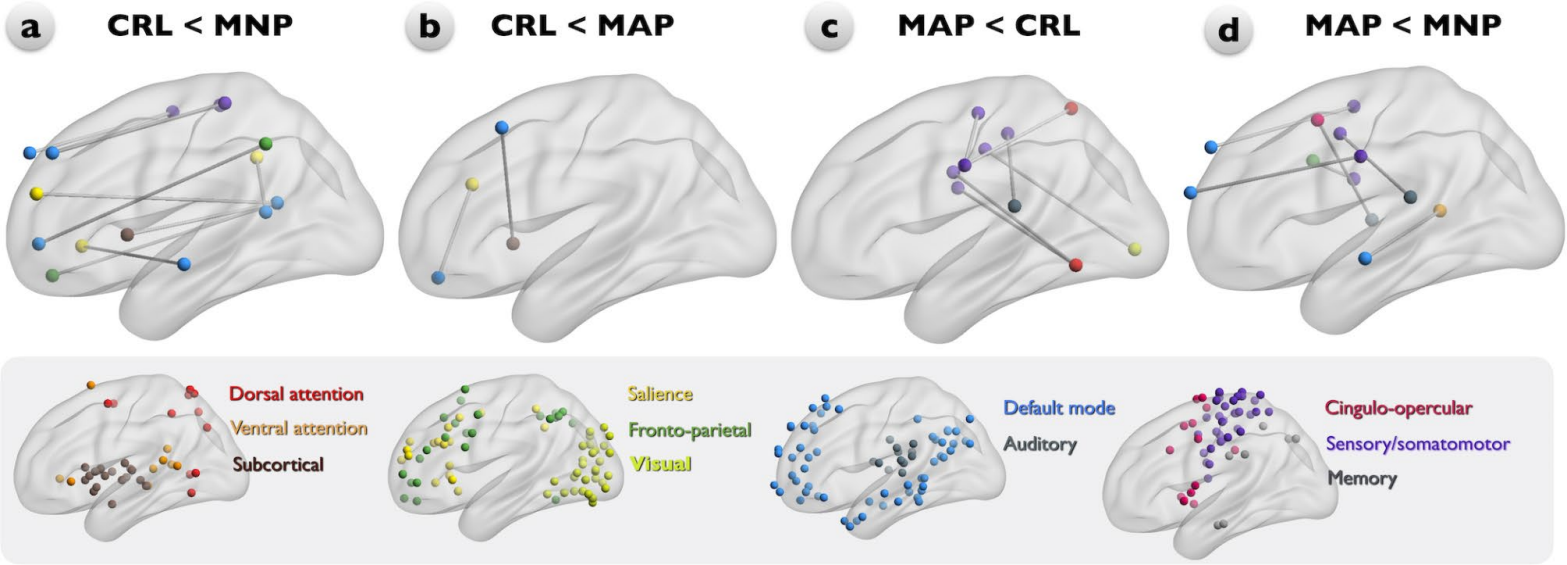
Significant group differences in large scale resting-state functional connectivity (rsFC) among the CRL, MNP, and MAP participant groups. Node color represents the network and corresponding distributions. **a**: Compared to the CRL group, the MNP subgroup shows enhanced rsFC between the FPN-DMN, SN-DMN, SMN-DMN, and SCN-DMN. **b-c**: The MAP group shows increased strength of rsFC between the SN-DMN and the SCN-DMN but decreased strength of rsFC between the SMN-DAN, SMN-VAN, SMN-SN, SMN-AN, and within SMN compared to the CRL group. **d**: Compared with the MNP subgroup, the MAP subgroup shows decreased strength of rsFC between the SMN-DMN, SMN-AN, SMN-FPN, DMN-VAN, and CON-AN. Abbreviations: CRL: healthy controls; MNP: methamphetamine users with no psychosis; MAP: methamphetamine-associated psychosis; DAN: dorsal attention network (red); VAN: ventral attention network (orange); SCN: subcortical network (maroon); SN: salience network (yellow); FPN: frontoparietal network (sea green); VN: visual network (lawn green); DMN: default mode network (dodger blue); AN: auditory network (slate grey); CON: cingulo-opercular network (deep pink); SMN: sensory/somatomotor network (dark violate); MN: memory network (grey)

Compared to the CRL group, the MNP subgroup showed enhanced rsFC between the FPN-DMN, SN-DMN, SMN-DMN, and SCN-DMN (Fig. 2a). The MAP subgroup also showed enhanced rsFC between the SN-DMN and the SCN-DMN compared to the CRL group (Fig. 2b**)** but decreased rsFC between the SMN-DAN, SMN-VAN, SMN-SN, SMN-AN (Fig. 2c). Additionally, compared to the MNP subgroup, the MAP subgroup exhibited a lower rsFC between the SMN-DMN, SMN-AN, SMN-FPN, DMN-VAN, and CON-AN (Fig. 2d). In intra-network analysis, decreased rsFC within SMN was observed in the MAP subgroup compared to the CRL group (Fig. 2c). After controlling for the nuisance factors of BDI and BAI, further link disruptions were observed between the DAN-FPN, FPN-VN, and SMN in the MAP subgroup compared to the MNP subgroup (Supplementary Fig. 1d). Compared to the CRL group, the MAP subgroup showed decreased rsFC only in the SMN-AN (Supplementary Fig. 1c). Additionally, the MNP subgroup exhibited increased rsFC between the VAN-SCN and SN-SMN, and within the FPN (Supplementary Fig. 1a).

### Relationships between functional connectivity and PANSS scores in individuals with MUD

**Fig. 3:**
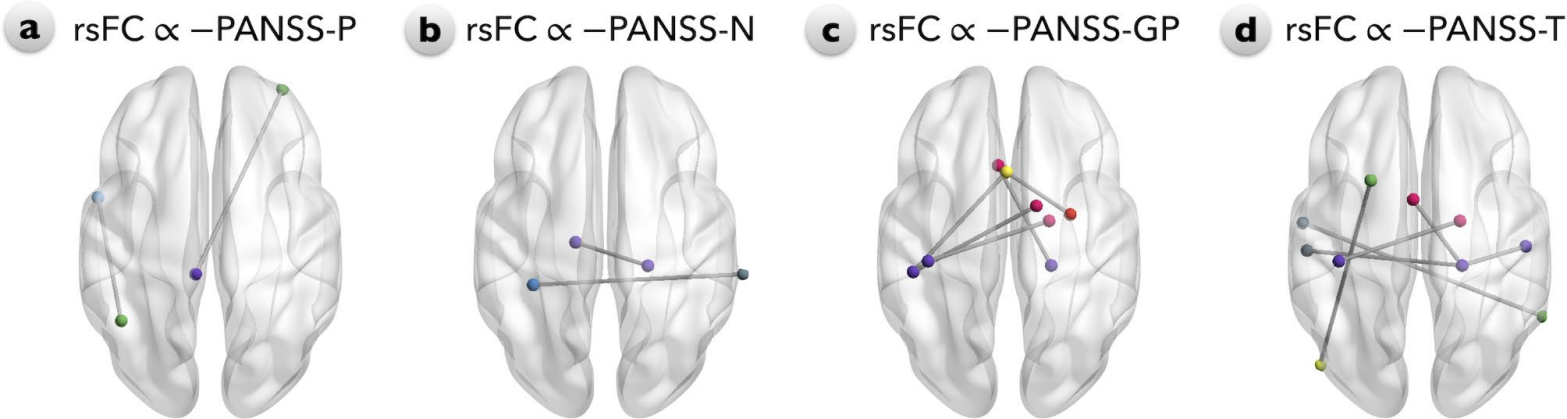
Relationships between and within the functional connectivity of the resting-state (rsFC) network and PANSS scores in the MAP and MNP subgroups. The PANSS-Total (PANSS-T), PANSS-Positive (PANSS-P), PANSS-Negative (PANSS-N), and PANSS-General Psychopathology (PANSS-GP) subscales were assessed, yielding a negative correlation with rsFC at corresponding sites. **a**: The PANSS-P is negatively correlated with the DMN-FPN and FPN-SMN. **b**: The PANSS-N is negatively correlated with the DMN-AN and within the SMN. **c**: The PANSS-GP is negatively correlated with the DAN-SN, SMN-CON, and SMN-SN. **d**: The PANSS-T is negatively correlated with the VN-FPN, AN-FPN, AN-SMN, SMN-CON, and within the SMN. Abbreviations: PANSS: Positive and Negative Syndrome Scale; DMN: default mode network (dodger blue); FPN: frontoparietal network (sea green); SMN: sensory/somatomotor network (dark violate); AN: auditory network (slate grey); DAN: dorsal attention network (red); SN: salience network (yellow); CON: cingulo-opercular network (deep pink); VN: visual network (lawn green)

A correlation analysis was conducted between both the inter-network and intra-network functional matrices of the two MUD subgroups and their corresponding psychotic severities, assessed using the PANSS-Total (PANSS-T), PANSS-Positive (PANSS-P), PANSS-Negative (PANSS-N) and PANSS-General Psychopathology (PANSS-GP) subscales. Every subscale showed a significant negative correlation with the rsFC of the corresponding site (Fig. 3). Specifically, PANSS-P was negatively correlated with the DMN-FPN, FPN-SMN (Fig. 3a), PANSS-N was negatively correlated with the DMN-AN and within SMN (Fig. 3b), PANSS-GP was negatively correlated with the DAN-SN, SMN-CON, SMN-SN (Fig. 3c), and PANSS-T was negatively correlated with the VN-FPN, AN-FPN, AN-SMN, SMN-CON and within the SMN (Fig. 3d). After adjusting for BDI and BAI, we found an additional negative correlation between SMN-AN connection and PANSS-P (Supplementary Fig. 2a). Additionally, the PANSS-GP exhibited negative correlations with both the FPN-AN and SCN-AN connections but showed no correlation with the DAN-SN and SMN-SN connections (Supplementary Fig. 2c). Moreover, an additional negative correlation between the PANSS-T and the SCN-DMN connection was identified (Supplementary Fig. 2d).

## Discussion

This study investigated differences in both inter-network and intra-network connectomes between the MNP and MAP subgroups and healthy controls. Our results showed that: (1) both of the MUD subgroups exhibited increases in rsFC between the SN-DMN and SCN-DMN compared to the CRL group; (2) the MNP subgroup exhibited an increase in rsFC strength between the FPN-DMN and SMN-DMN compared to the CRL group; (3) the MAP subgroup exhibited a decrease in rsFC strength between the SMN-DAN, SMN-VAN, SMN-SN, SMN-AN and within the SMN compared to the CRL group; (4) compared to the MNP subgroup, the MAP subgroup exhibited a lower rsFC between the SMN-DMN, SMN-AN, SMN-FPN, DMN-VAN, and CON-AN; and (5) PANSS was negatively correlated with the rsFC in both MUD subgroups. While PANSS-P was negatively correlated with the DMN-FPN and FPN-SMN, PANSS-N was negatively correlated with the DMN-AN and within the SMN. These results lend support to the hypothesis that certain network dysfunctions represent a core neural deficit underlying psychosis development in individuals with MUD.

### Inter-network connectivity in METH users: Alterations in DMN-SN-FPN coupling

The findings of this study appear consistent with the model of disrupted DMN–SN–FPN coupling in substance use disorders [20, 42–44]. The FPN is related to cognitive control and executive function, especially for external stimuli, while the DMN is associated with self-referential cognition [20]. Cooperation between the DMN and FPN produces an internal train of thought [45]. Furthermore, the FPN regulates the two anticorrelated behaviors between the DMN and the CCN-DAN. It coordinates dynamic and task-dependent switching between the CCN-DAN and DMN in response to various task demands, given its anatomical location in the parietal lobe and its highest degree of internetwork temporal variability [20, 46, 47]. Our results show increased rsFC between the FPN and DMN in the MNP subgroup compared to controls. This finding is consistent with the result found in the previous literature [20], which suggests that chronic METH exposure is related to a disability of the FPN in coactivation of temporal variability and a switch between the DMN and the CCN-DAN, resulting in hyperconnectivity of the FPN-DMN. However, we did not find hyperconnectivity in the FPN-DMN in the MAP subgroup compared to the CRL group. This disparity could be the result of heterogeneity in the duration of psychotic symptoms among members of the MAP subgroup in this study.

Increased rsFC strength between the SN-DMN was found in both MUD subgroups compared to the CRL group, supporting several other studies [42–44]. Briefly, the SN, consisting of the anterior insula and the dorsal anterior cingulate cortex, detects significant events and facilitates other networks, modulating autonomic reactivity in response to significant stimuli [48]. Li et al. showed increased functional connectivity between the posterior cingulate cortex (PCC) and the insula in chronic heroin users compared to healthy controls, and they showed a positive correlation with the duration of heroin use [43]. Similarly, Wetherill et al. demonstrated enhanced functional connectivity of the PCC-right anterior insula in cannabis-dependent individuals compared to healthy controls [44]. Naqvi and Bechara addressed the role of the insula in the process of interoceptive effects of drug use and drug urges [49, 50]. Zhang and Volkow found that altered SN-DMN connectivity might enhance salience toward drug signals [42]. Collectively, these studies suggest that altered rsFC in the SN-DMN could be a factor in the increased relevance of METH use, and it could ultimately contribute to METH addiction and relapse.

### Aberrant inter-network rsFC of SMN and DMN in the MNP and MAP subgroups

We demonstrated a disrupted rsFC in functional networks that interact with the SMN in the MUD group. The MNP subgroup showed enhanced rsFC in the SMN-DMN compared to the CRL group. The MAP subgroup, however, showed decreased rsFC in inter-SMN networks (e.g., SMN-DAN, SMN-VAN, SMN-SN, and SMN-AN) compared to the CRL group. In addition, the MAP subgroup showed lower rsFC in the SMN-DMN compared to the MNP subgroup. Aberrant inter-network rsFC of the SMN in METH users has been described in the literature. For example, Li et al. reported lower rsFC between the SMN and the cerebellum in individuals with MUD compared to healthy controls [51] and suggested that these impaired networks were related to drug compulsivity and the inability to control habitual drug consumption.

The DMN can be divided into anterior and posterior regions. The anterior DMN plays a role in personal and emotional regulation, and the posterior DMN directs attention to internal thoughts [42]. The two regions coordinate with each other, with the posterior DMN directing attention to internal/external stimuli and transferring the stimuli to the anterior DMN, which monitors the internal states and regulates emotional and personal relevance [42, 52–54]. A review of the literature on DMN dysfunction in addiction found that rsFC in addicted individuals decreased in the anterior DMN and increased in the posterior DMN [42]. Additionally, dysfunction of the ventromedial prefrontal cortex, which is part of the anterior DMN, is correlated with compromised self-awareness in drug addiction, including self-reported behavior dissociation and inappropriate social behavior [54].

Taken together, our results potentially imply that MAP is distinct from MNP in terms of aberrant inter-network rsFC of the SMN, and we suggest that those with MAP are more vulnerable to drug-related neurobiological deficits and subsequent psychological consequences such as greater compulsivity and preserving less self-awareness relative to those with MNP. Notably, all METH users were scanned after discontinuation of METH use for at least 3–4 weeks, by which time the psychotic symptoms had generally subsided. The distinct connectome profiles between the MNP and MAP subgroups suggest that MAP is associated with more specific neurobiological pathways in susceptibility to psychosis development among individuals exposed to METH. Identification of these altered networks could aid in future classification approaches.

### Relationships between functional connectivity and PANSS scores in individuals with MUD

This study shows that the severity of positive psychotic symptoms, as measured by the PANSS-P, is negatively correlated with the strength of functional connectivity in the FPN-DMN among the MNP and MAP. The FPN plays a vital role in cognitive control and executive function and acts as a flexible hub in adaptive implementations of task demands [55]. Cognitive deficits or impairments are common in psychotic disorders, and altered FPN has been associated with psychotic disorders [56]. Lewandowski et al. showed an additional reduction of FPN connectivity in psychotic patients with impaired cognition compared to psychotic patients with intact cognition [57]. Notably, recent studies have shown damage to the FPN-DMN in substance use disorders. Li et al. reported that hypoconnectivity between the left executive control network (ECN) and the DMN is associated with heroin relapse behavior [58]. Liang et al. showed that cocaine addiction is associated with disrupted connectivity between the PCC and the ECN [59]. In particular, our result is supported by Gong et al. who demonstrated a decrease of FPN-DMN in METH abstainers [60]. Taken together, our findings suggest that hypoconnectivity between the FPN-DMN is positively related to the severity of positive symptoms.

Our results also demonstrate that PANSS-N is negatively correlated with rsFC in the DMN-AN and intra-SMN. The control of emotions and behaviors is regulated by the anterior DMN, whereas the AN interacts with the external environment and further processes auditory information and cognitive tasks such as language [61]. The negative symptoms of PANSS include blunted affect, emotional withdrawal, poor rapport, passive/apathetic social withdrawal, difficulty in abstract thinking, lack of spontaneity and flow of conversation, and stereotyped thinking. Our findings suggest that these negative symptoms are related to decreased activity caused by external or environmental stimuli. Correspondingly, the observed hypo-rsFC between the DMN-AN could indicate a slower reaction or a delayed response in the attentional processing of auditory information and social cognition. Subsequently, these negative symptoms may exert an influence on the SMN domain.

In this study, both the MNP and MAP subgroups exhibited a negative correlation between PANSS-GP and the rsFC of the CON-SMN, DAN-SN, and SMN-SN. In addition to FPN, the CON regulates top-down control of executive functioning. On the other hand, the DAN is thought to be involved in attending to external stimuli and engaging in an active attention process such as visuospatial attention during a task [62]. The DAN is also suggested to involve top-down attention to sensory inputs [62]. Therefore, decreased rsFC involving the DAN and the CON could explain executive dysfunction in MNP and MAP.

### Limitations

This study has several limitations. Firstly, its cross-sectional design does not allow investigation of changes in the functional connectome over time, for example, from initial recreational use of METH to dependence, or to assess reversibility potential after long-term abstinence. In other words, the single-observation nature of the study precludes the possibility of confirming a causal relationship. Secondly, our MAP subgroup exhibited more severe depression and anxiety than our MNP subgroup. This is in line with previous evidence indicating that METH users with symptoms of anxiety and depression had a significantly greater risk of experiencing psychosis [63]. Our study, which focused on a single psychiatric phenomenon (psychosis), could conceal the existence of significant overlap in the distributions of connectome functioning related to subthreshold depression and anxiety symptoms. This could consequently create an illusion of group specificity for psychosis based on our connectome finding when, in reality, there could be shared and coordinated functional neural networks among complex psychiatric conditions [10, 64]. However, after controlling for BDI and BAI factors, we observed more disrupted inter-network rsFC between the MAP and MNP subgroups. These results further highlighted the credibility of the different neurobiological pathways associated with MAP compared to MNP, as proposed in our study. The third limitation of our study is that all recruited participants were from a single center, with a small sample size consisting exclusively of men; therefore, our results cannot represent all individuals who use METH and, specifically, it cannot represent the female population. Given the relatively infrequent occurrence of MAP compared to other substance use disorders, conduct of a formal sample size calculation was not feasible. Therefore, all eligible patients who were identified during the study period were recruited. A larger dataset from a gender-balanced population is warranted in future studies. A fourth limitation is that the potential effects of nicotine on altered network connections cannot be entirely excluded. Previous studies have shown that nicotine decreases activity within the DMN during the resting state [65] and that chronic nicotine exposure decreases the effectiveness of the functional network, while acute nicotine exposure enhances connectivity of particular limbic circuits [66]. Fifth, we performed rs-MRI after the participants in the MUD group were free from withdrawal symptoms, which limits our ability to generalize the data to those in an acute psychotic state, which usually subsides within one week after METH use [7, 8].

## Conclusions

In summary, this study shows that exposure to METH alters global functional connectivity in the DMN, FPN, SN, and SMN. In addition, the severity of psychotic symptoms is inversely correlated with the rsFC of the corresponding sites, and the PANSS-P score is negatively correlated with the DMN-FPN and FPN-SMN. Moreover, the MAP subgroup exhibited more disrupted rsFC in the DMN and SMN than the MNP subgroup, suggesting that MAP involves greater drug compulsivity and less self-awareness. Our results suggest that MAP involves specific neurobiological pathways that determine the greater vulnerability of some METH users to developing psychosis. Identifying these modified networks could be beneficial for future classification strategies aimed at distinguishing individuals with and without psychoses.

## Electronic supplementary material

Below is the link to the electronic supplementary material.

Supplementary Material 1: Fig. 1: The upper panels showed significant group differences in resting-state functional connectivity (rsFC) between the CRL, MNP, and MAP groups without controlling for BDI and BAI factors. The lower panels demonstrated various rsFC after additional controlling for both BDI and BAI factors. **a**: Compared to the CRL group, the MNP subgroup exhibited additionally increased rsFC between the VAN-SCN, SN-SMN, and within the FPN-FPN. **b**: The MAP subgroup exhibited no changes in enhanced rsFC of SN-DMN and SCN-DMN compared to the CRL group. **c**: the MAP subgroup only showed decreased rsFC between the SMN-AN compared to the CRL group. **d**: More disrupted rsFC between the DAN-FPN, FPN-VN, and within the SMN-SMN in the MAP subgroup compared to the MNP subgroup. Abbreviations: CRL: healthy controls; FD: frame displacement; MNP: methamphetamine users with no psychosis; MAP: methamphetamine-associated psychosis; DAN: dorsal attention network (red); VAN: ventral attention network (orange); SCN: subcortical network (maroon); SN: salience network (yellow); FPN: frontoparietal network (sea green); VN: visual network (lawn green); DMN: default mode network (dodger blue); AN: auditory network (slate grey); CON: cingulo-opercular network (deep pink); SMN: sensory/somatomotor network (dark violate); MN: memory network (grey). Fig. 2: The upper panels showed the relationships between and within the functional connectivity of the resting-state (rsFC) network and PANSS scores in the MAP and MNP subgroups without controlling for BDI and BAI factors. The lower panel demonstrated various rsFC after adjusting for BDI and BAI. **a**: An additional negative correlation between the SMN-AN and the PANSS-P score. **b**: No changes in the negative correlation for the PANSS-N and rsFC of DMN-AN and within SMN-SMN. **c**: Additionally, the PANSS-GP was negatively correlated with the FPN-AN and SCN-AN, but showed no correlation with the DAN-SN and SMN-SN. **d**: An additional negative correlation between the PANSS-T and the SCN-DMN connection was identified. Abbreviations: FD: frame displacement; MAP: methamphetamine-associated psychosis; MNP: methamphetamine users with no psychosis; PANSS: Positive and Negative Syndrome Scale; DMN: default mode network (dodger blue); FPN: frontoparietal network (sea green); SMN: sensory/somatomotor network (dark violate); AN: auditory network (slate grey); DAN: dorsal attention network (red); SN: salience network (yellow); CON: cingulo-opercular network (deep pink); VN: visual network (lawn green). Table 1: The MNI coordinates and suggested networks of 245 ROIs.

## Data Availability

Data supporting our findings in this study are available upon request from the corresponding author. Data are not publicly available because of privacy or ethical restrictions.
