# Supplementary material for: The distinct functional brain network and its association with psychotic symptom severity in men with methamphetamine-associated psychosis
Source: BMC Psychiatry. 2024 Oct 10;24:671. doi: 10.1186/s12888-024-06112-4 (PMC11468263; doi:10.1186/s12888-024-06112-4)
Supplement: Supplementary file 1 — Supplementary Material 1: Fig. 1: The upper panels showed significant group differences in resting-state functional connectivity (rsFC) between the CRL, MNP, and MAP groups without controlling for BDI and BAI factors. The lower panels demonstrated various rsFC after additional controlling for both BDI and BAI factors. a: Compared to the CRL group, the MNP subgroup exhibited additionally increased rsFC between the VAN-SCN, SN-SMN, and within the FPN-FPN. b: The MAP subgroup exhibited no changes in enhanced rsFC of SN-DMN and SCN-DMN compared to the CRL group. c: the MAP subgroup only showed decreased rsFC between the SMN-AN compared to the CRL group. d: More disrupted rsFC between the DAN-FPN, FPN-VN, and within the SMN-SMN in the MAP subgroup compared to the MNP subgroup. Abbreviations: CRL: healthy controls; FD: frame displacement; MNP: methamphetamine users with no psychosis; MAP: methamphetamine-associated psychosis; DAN: dorsal attention network (red); VAN: ventral attention network (orange); SCN: subcortical network (maroon); SN: salience network (yellow); FPN: frontoparietal network (sea green); VN: visual network (lawn green); DMN: default mode network (dodger blue); AN: auditory network (slate grey); CON: cingulo-opercular network (deep pink); SMN: sensory/somatomotor network (dark violate); MN: memory network (grey). Fig. 2: The upper panels showed the relationships between and within the functional connectivity of the resting-state (rsFC) network and PANSS scores in the MAP and MNP subgroups without controlling for BDI and BAI factors. The lower panel demonstrated various rsFC after adjusting for BDI and BAI. a: An additional negative correlation between the SMN-AN and the PANSS-P score. b: No changes in the negative correlation for the PANSS-N and rsFC of DMN-AN and within SMN-SMN. c: Additionally, the PANSS-GP was negatively correlated with the FPN-AN and SCN-AN, but showed no correlation with the DAN-SN and SMN-SN. d: An additional negative correlation [file 12888_2024_6112_MOESM1_ESM.docx]

**
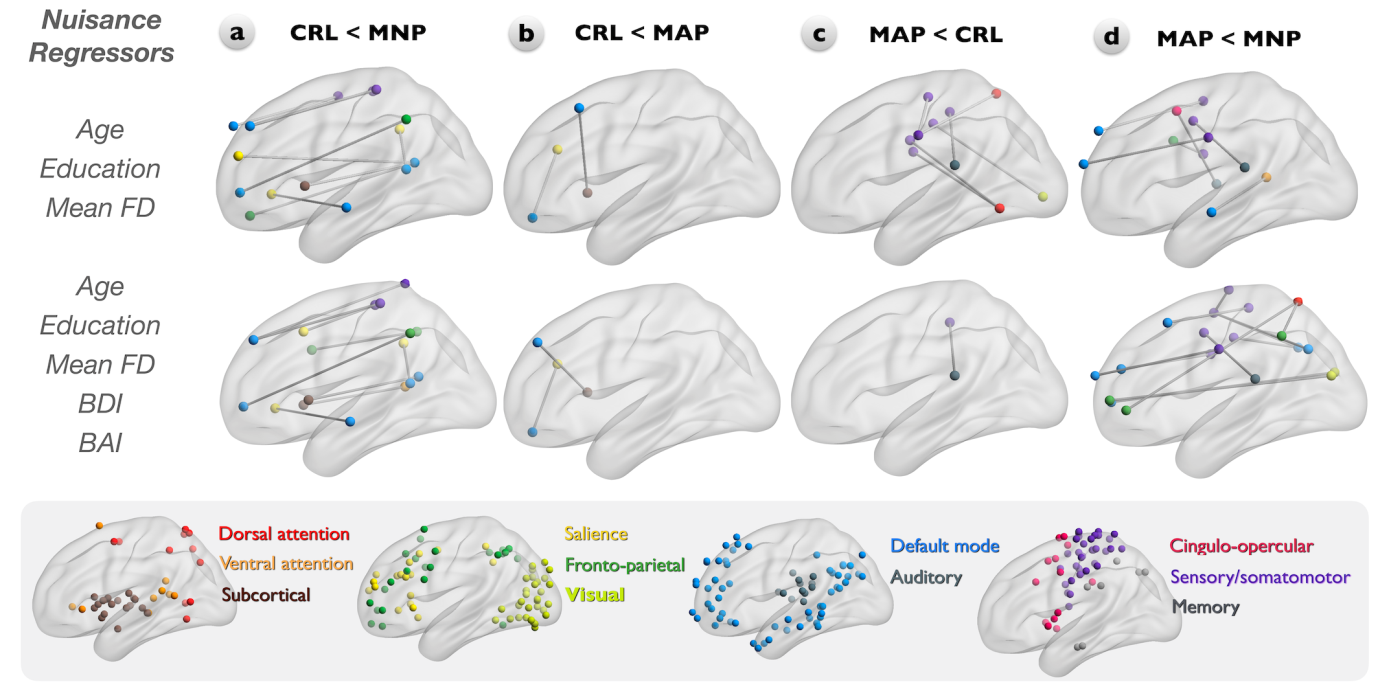
**

**Supplementary Figure 1**: The upper panels showed significant group differences in resting-state functional connectivity (rsFC) between the CRL, MNP, and MAP groups without controlling for BDI and BAI factors. The lower panels demonstrated various rsFC after additional controlling for both BDI and BAI factors. **a:** Compared to the CRL group, the MNP subgroup exhibited additionally increased rsFC between the VAN-SCN, SN-SMN, and within the FPN-FPN. **b:** The MAP subgroup exhibited no changes in enhanced rsFC of SN-DMN and SCN-DMN compared to the CRL group. **c:** the MAP subgroup only showed decreased rsFC between the SMN-AN compared to the CRL group. **d:** More disrupted rsFC between the DAN-FPN, FPN-VN, and within the SMN-SMN in the MAP subgroup compared to the MNP subgroup. Abbreviations: CRL: healthy controls; FD: frame displacement; MNP: methamphetamine users with no psychosis; MAP: methamphetamine-associated psychosis; DAN: dorsal attention network (red); VAN: ventral attention network (orange); SCN: subcortical network (maroon); SN: salience network (yellow); FPN: frontoparietal network (sea green); VN: visual network (lawn green); DMN: default mode network (dodger blue); AN: auditory network (slate grey); CON: cingulo-opercular network (deep pink); SMN: sensory/somatomotor network (dark violate); MN: memory network (grey).


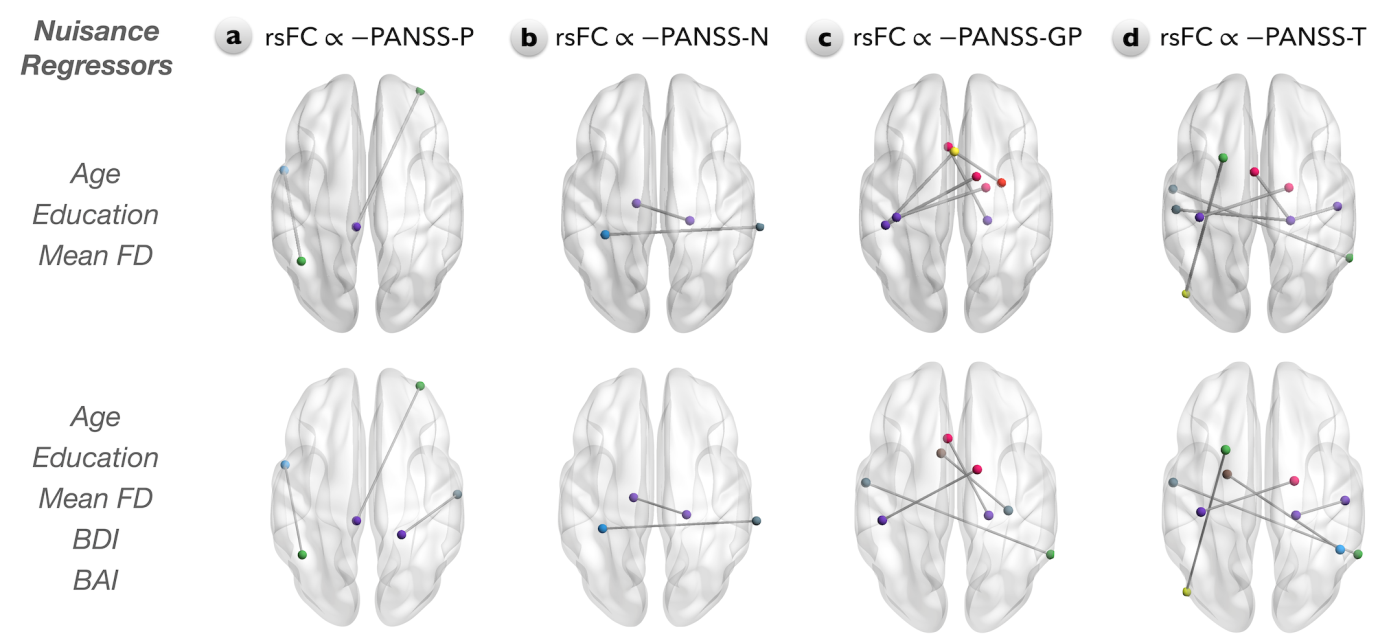


**Supplementary Figure 2:** The upper panels showed the relationships between and within the functional connectivity of the resting-state (rsFC) network and PANSS scores in the MAP and MNP subgroups without controlling for BDI and BAI factors. The lower panel demonstrated various rsFC after adjusting for BDI and BAI. **a:** An additional negative correlation between the SMN-AN and the PANSS-P score. **b:** No changes in the negative correlation for the PANSS-N and rsFC of DMN-AN and within SMN-SMN. **c:** Additionally, the PANSS-GP was negatively correlated with the FPN-AN and SCN-AN, but showed no correlation with the DAN-SN and SMN-SN. **d:** An additional negative correlation between the PANSS-T and the SCN-DMN connection was identified. Abbreviations: FD: frame displacement; MAP: methamphetamine-associated psychosis; MNP: methamphetamine users with no psychosis; PANSS: Positive and Negative Syndrome Scale; DMN: default mode network (dodger blue); FPN: frontoparietal network (sea green); SMN: sensory/somatomotor network (dark violate); AN: auditory network (slate grey); DAN: dorsal attention network (red); SN: salience network (yellow); CON: cingulo-opercular network (deep pink); VN: visual network (lawn green).

**Supplementary Tables**

**Supplementary Table 1: The MNI coordinates and suggested networks of 245 ROIs**

| ROI | MNI Coordinate | | | Suggested network | Source |
| --- | --- | --- | --- | --- | --- |
|  | X | Y | Z |  |  |
| 1 | 10 | -62 | 61 | Dorsal Attention | Power et al., 2012 |
| 2 | -52 | -63 | 5 | Dorsal Attention | Power et al., 2012 |
| 3 | 22 | -65 | 48 | Dorsal Attention | Power et al., 2012 |
| 4 | 46 | -59 | 4 | Dorsal Attention | Power et al., 2012 |
| 5 | 25 | -58 | 60 | Dorsal Attention | Power et al., 2012 |
| 6 | -33 | -46 | 47 | Dorsal Attention | Power et al., 2012 |
| 7 | -27 | -71 | 37 | Dorsal Attention | Power et al., 2012 |
| 8 | -32 | -1 | 54 | Dorsal Attention | Power et al., 2012 |
| 9 | -42 | -60 | -9 | Dorsal Attention | Power et al., 2012 |
| 10 | -17 | -59 | 64 | Dorsal Attention | Power et al., 2012 |
| 11 | 29 | -5 | 54 | Dorsal Attention | Power et al., 2012 |
| 12 | -10 | 11 | 67 | Dorsal Attention | Power et al., 2012 |
| 13 | 54 | -43 | 22 | Dorsal Attention | Power et al., 2012 |
| 14 | -56 | -50 | 10 | Dorsal Attention | Power et al., 2012 |
| 15 | -55 | -40 | 14 | Dorsal Attention | Power et al., 2012 |
| 16 | 52 | -33 | 8 | Dorsal Attention | Power et al., 2012 |
| 17 | 51 | -29 | -4 | Dorsal Attention | Power et al., 2012 |
| 18 | 56 | -46 | 11 | Dorsal Attention | Power et al., 2012 |
| 19 | 53 | 33 | 1 | Dorsal Attention | Power et al., 2012 |
| 20 | -49 | 25 | -1 | Dorsal Attention | Power et al., 2012 |
| 21 | 6 | -24 | 0 | Subcortical | Power et al., 2012 |
| 22 | -2 | -13 | 12 | Subcortical | Power et al., 2012 |
| 23 | -10 | -18 | 7 | Subcortical | Power et al., 2012 |
| 24 | 12 | -17 | 8 | Subcortical | Power et al., 2012 |
| 25 | -5 | -28 | -4 | Subcortical | Power et al., 2012 |
| 26 | -22 | 7 | -5 | Subcortical | Power et al., 2012 |
| 27 | -15 | 4 | 8 | Subcortical | Power et al., 2012 |
| 28 | 31 | -14 | 2 | Subcortical | Power et al., 2012 |
| 29 | -31 | -11 | 0 | Subcortical | Power et al., 2012 |
| 30 | 15 | 5 | 7 | Subcortical | Power et al., 2012 |
| 31 | 9 | -4 | 6 | Subcortical | Power et al., 2012 |
| 32 | 9 | 9 | -8 | Subcortical | Martino et al., 2008 |
| 33 | -9 | 9 | -8 | Subcortical | Martino et al., 2009 |
| 34 | 10 | 15 | 0 | Subcortical | Martino et al., 2010 |
| 35 | -10 | 15 | 0 | Subcortical | Martino et al., 2011 |
| 36 | 13 | 15 | 9 | Subcortical | Martino et al., 2012 |
| 37 | -13 | 15 | 9 | Subcortical | Martino et al., 2013 |
| 38 | 28 | 1 | 3 | Subcortical | Martino et al., 2014 |
| 39 | -28 | 1 | 3 | Subcortical | Martino et al., 2015 |
| 40 | 25 | 8 | 6 | Subcortical | Martino et al., 2016 |
| 41 | -25 | 8 | 6 | Subcortical | Martino et al., 2017 |
| 42 | 20 | 12 | 3 | Subcortical | Martino et al., 2018 |
| 43 | -20 | 12 | 3 | Subcortical | Martino et al., 2019 |
| 44 | 24 | -4 | -17 | Subcortical | Yarkoni et al., 2011 |
| 45 | -22 | -4 | -17 | Subcortical | Yarkoni et al., 2012 |
| 46 | 11 | -39 | 50 | Salience | Power et al., 2012 |
| 47 | 55 | -45 | 37 | Salience | Power et al., 2012 |
| 48 | 42 | 0 | 47 | Salience | Power et al., 2012 |
| 49 | 31 | 33 | 26 | Salience | Power et al., 2012 |
| 50 | 48 | 22 | 10 | Salience | Power et al., 2012 |
| 51 | -35 | 20 | 0 | Salience | Power et al., 2012 |
| 52 | 36 | 22 | 3 | Salience | Power et al., 2012 |
| 53 | 37 | 32 | -2 | Salience | Power et al., 2012 |
| 54 | 34 | 16 | -8 | Salience | Power et al., 2012 |
| 55 | -11 | 26 | 25 | Salience | Power et al., 2012 |
| 56 | -1 | 15 | 44 | Salience | Power et al., 2012 |
| 57 | -28 | 52 | 21 | Salience | Power et al., 2012 |
| 58 | 0 | 30 | 27 | Salience | Power et al., 2012 |
| 59 | 5 | 23 | 37 | Salience | Power et al., 2012 |
| 60 | 10 | 22 | 27 | Salience | Power et al., 2012 |
| 61 | 31 | 56 | 14 | Salience | Power et al., 2012 |
| 62 | 26 | 50 | 27 | Salience | Power et al., 2012 |
| 63 | -39 | 51 | 17 | Salience | Power et al., 2012 |
| 64 | -44 | 2 | 46 | Fronto-parietal | Power et al., 2012 |
| 65 | 48 | 25 | 27 | Fronto-parietal | Power et al., 2012 |
| 66 | -47 | 11 | 23 | Fronto-parietal | Power et al., 2012 |
| 67 | -53 | -49 | 43 | Fronto-parietal | Power et al., 2012 |
| 68 | -23 | 11 | 64 | Fronto-parietal | Power et al., 2012 |
| 69 | 58 | -53 | -14 | Fronto-parietal | Power et al., 2012 |
| 70 | 24 | 45 | -15 | Fronto-parietal | Power et al., 2012 |
| 71 | 34 | 54 | -13 | Fronto-parietal | Power et al., 2012 |
| 72 | 47 | 10 | 33 | Fronto-parietal | Power et al., 2012 |
| 73 | -41 | 6 | 33 | Fronto-parietal | Power et al., 2012 |
| 74 | -42 | 38 | 21 | Fronto-parietal | Power et al., 2012 |
| 75 | 38 | 43 | 15 | Fronto-parietal | Power et al., 2012 |
| 76 | 49 | -42 | 45 | Fronto-parietal | Power et al., 2012 |
| 77 | -28 | -58 | 48 | Fronto-parietal | Power et al., 2012 |
| 78 | 44 | -53 | 47 | Fronto-parietal | Power et al., 2012 |
| 79 | 32 | 14 | 56 | Fronto-parietal | Power et al., 2012 |
| 80 | 37 | -65 | 40 | Fronto-parietal | Power et al., 2012 |
| 81 | -42 | -55 | 45 | Fronto-parietal | Power et al., 2012 |
| 82 | 40 | 18 | 40 | Fronto-parietal | Power et al., 2012 |
| 83 | -34 | 55 | 4 | Fronto-parietal | Power et al., 2012 |
| 84 | -42 | 45 | -2 | Fronto-parietal | Power et al., 2012 |
| 85 | 33 | -53 | 44 | Fronto-parietal | Power et al., 2012 |
| 86 | 43 | 49 | -2 | Fronto-parietal | Power et al., 2012 |
| 87 | -42 | 25 | 30 | Fronto-parietal | Power et al., 2012 |
| 88 | -3 | 26 | 44 | Fronto-parietal | Power et al., 2012 |
| 89 | 18 | -47 | -10 | Visual | Power et al., 2012 |
| 90 | 40 | -72 | 14 | Visual | Power et al., 2012 |
| 91 | 8 | -72 | 11 | Visual | Power et al., 2012 |
| 92 | -8 | -81 | 7 | Visual | Power et al., 2012 |
| 93 | -28 | -79 | 19 | Visual | Power et al., 2012 |
| 94 | 20 | -66 | 2 | Visual | Power et al., 2012 |
| 95 | -24 | -91 | 19 | Visual | Power et al., 2012 |
| 96 | 27 | -59 | -9 | Visual | Power et al., 2012 |
| 97 | -15 | -72 | -8 | Visual | Power et al., 2012 |
| 98 | -18 | -68 | 5 | Visual | Power et al., 2012 |
| 99 | 43 | -78 | -12 | Visual | Power et al., 2012 |
| 100 | -47 | -76 | -10 | Visual | Power et al., 2012 |
| 101 | -14 | -91 | 31 | Visual | Power et al., 2012 |
| 102 | 15 | -87 | 37 | Visual | Power et al., 2012 |
| 103 | 29 | -77 | 25 | Visual | Power et al., 2012 |
| 104 | 20 | -86 | -2 | Visual | Power et al., 2012 |
| 105 | 15 | -77 | 31 | Visual | Power et al., 2012 |
| 106 | -16 | -52 | -1 | Visual | Power et al., 2012 |
| 107 | 42 | -66 | -8 | Visual | Power et al., 2012 |
| 108 | 24 | -87 | 24 | Visual | Power et al., 2012 |
| 109 | 6 | -72 | 24 | Visual | Power et al., 2012 |
| 110 | -42 | -74 | 0 | Visual | Power et al., 2012 |
| 111 | 26 | -79 | -16 | Visual | Power et al., 2012 |
| 112 | -16 | -77 | 34 | Visual | Power et al., 2012 |
| 113 | -3 | -81 | 21 | Visual | Power et al., 2012 |
| 114 | -40 | -88 | -6 | Visual | Power et al., 2012 |
| 115 | 37 | -84 | 13 | Visual | Power et al., 2012 |
| 116 | 6 | -81 | 6 | Visual | Power et al., 2012 |
| 117 | -26 | -90 | 3 | Visual | Power et al., 2012 |
| 118 | -33 | -79 | -13 | Visual | Power et al., 2012 |
| 119 | 37 | -81 | 1 | Visual | Power et al., 2012 |
| 120 | -41 | -75 | 26 | Default mode | Power et al., 2012 |
| 121 | 6 | 67 | -4 | Default mode | Power et al., 2012 |
| 122 | 8 | 48 | -15 | Default mode | Power et al., 2012 |
| 123 | -13 | -40 | 1 | Default mode | Power et al., 2012 |
| 124 | -18 | 63 | -9 | Default mode | Power et al., 2012 |
| 125 | -46 | -61 | 21 | Default mode | Power et al., 2012 |
| 126 | 43 | -72 | 28 | Default mode | Power et al., 2012 |
| 127 | -44 | 12 | -34 | Default mode | Power et al., 2012 |
| 128 | 46 | 16 | -30 | Default mode | Power et al., 2012 |
| 129 | -68 | -23 | -16 | Default mode | Power et al., 2012 |
| 130 | -44 | -65 | 35 | Default mode | Power et al., 2012 |
| 131 | -39 | -75 | 44 | Default mode | Power et al., 2012 |
| 132 | -7 | -55 | 27 | Default mode | Power et al., 2012 |
| 133 | 6 | -59 | 35 | Default mode | Power et al., 2012 |
| 134 | -11 | -56 | 16 | Default mode | Power et al., 2012 |
| 135 | -3 | -49 | 13 | Default mode | Power et al., 2012 |
| 136 | 8 | -48 | 31 | Default mode | Power et al., 2012 |
| 137 | 15 | -63 | 26 | Default mode | Power et al., 2012 |
| 138 | -2 | -37 | 44 | Default mode | Power et al., 2012 |
| 139 | 11 | -54 | 17 | Default mode | Power et al., 2012 |
| 140 | 52 | -59 | 36 | Default mode | Power et al., 2012 |
| 141 | 23 | 33 | 48 | Default mode | Power et al., 2012 |
| 142 | -10 | 39 | 52 | Default mode | Power et al., 2012 |
| 143 | -16 | 29 | 53 | Default mode | Power et al., 2012 |
| 144 | -35 | 20 | 51 | Default mode | Power et al., 2012 |
| 145 | 22 | 39 | 39 | Default mode | Power et al., 2012 |
| 146 | 13 | 55 | 38 | Default mode | Power et al., 2012 |
| 147 | -10 | 55 | 39 | Default mode | Power et al., 2012 |
| 148 | -20 | 45 | 39 | Default mode | Power et al., 2012 |
| 149 | 6 | 54 | 16 | Default mode | Power et al., 2012 |
| 150 | 6 | 64 | 22 | Default mode | Power et al., 2012 |
| 151 | -7 | 51 | -1 | Default mode | Power et al., 2012 |
| 152 | 9 | 54 | 3 | Default mode | Power et al., 2012 |
| 153 | -3 | 44 | -9 | Default mode | Power et al., 2012 |
| 154 | 8 | 42 | -5 | Default mode | Power et al., 2012 |
| 155 | -11 | 45 | 8 | Default mode | Power et al., 2012 |
| 156 | -2 | 38 | 36 | Default mode | Power et al., 2012 |
| 157 | -3 | 42 | 16 | Default mode | Power et al., 2012 |
| 158 | -20 | 64 | 19 | Default mode | Power et al., 2012 |
| 159 | -8 | 48 | 23 | Default mode | Power et al., 2012 |
| 160 | 65 | -12 | -19 | Default mode | Power et al., 2012 |
| 161 | -56 | -13 | -10 | Default mode | Power et al., 2012 |
| 162 | -58 | -30 | -4 | Default mode | Power et al., 2012 |
| 163 | 65 | -31 | -9 | Default mode | Power et al., 2012 |
| 164 | -68 | -41 | -5 | Default mode | Power et al., 2012 |
| 165 | 13 | 30 | 59 | Default mode | Power et al., 2012 |
| 166 | 12 | 36 | 20 | Default mode | Power et al., 2012 |
| 167 | 52 | -2 | -16 | Default mode | Power et al., 2012 |
| 168 | -26 | -40 | -8 | Default mode | Power et al., 2012 |
| 169 | 27 | -37 | -13 | Default mode | Power et al., 2012 |
| 170 | -34 | -38 | -16 | Default mode | Power et al., 2012 |
| 171 | 52 | 7 | -30 | Default mode | Power et al., 2012 |
| 172 | -53 | 3 | -27 | Default mode | Power et al., 2012 |
| 173 | 47 | -50 | 29 | Default mode | Power et al., 2012 |
| 174 | -49 | -42 | 1 | Default mode | Power et al., 2012 |
| 175 | -46 | 31 | -13 | Default mode | Power et al., 2012 |
| 176 | 49 | 35 | -12 | Default mode | Power et al., 2012 |
| 177 | 32 | -26 | 13 | Auditory | Power et al., 2012 |
| 178 | 65 | -33 | 20 | Auditory | Power et al., 2012 |
| 179 | 58 | -16 | 7 | Auditory | Power et al., 2012 |
| 180 | -38 | -33 | 17 | Auditory | Power et al., 2012 |
| 181 | -60 | -25 | 14 | Auditory | Power et al., 2012 |
| 182 | -49 | -26 | 5 | Auditory | Power et al., 2012 |
| 183 | 43 | -23 | 20 | Auditory | Power et al., 2012 |
| 184 | -50 | -34 | 26 | Auditory | Power et al., 2012 |
| 185 | -53 | -22 | 23 | Auditory | Power et al., 2012 |
| 186 | -55 | -9 | 12 | Auditory | Power et al., 2012 |
| 187 | 56 | -5 | 13 | Auditory | Power et al., 2012 |
| 188 | 59 | -17 | 29 | Auditory | Power et al., 2012 |
| 189 | -30 | -27 | 12 | Auditory | Power et al., 2012 |
| 190 | -3 | 2 | 53 | Cingulo-opercular | Power et al., 2012 |
| 191 | 54 | -28 | 34 | Cingulo-opercular | Power et al., 2012 |
| 192 | 19 | -8 | 64 | Cingulo-opercular | Power et al., 2012 |
| 193 | -16 | -5 | 71 | Cingulo-opercular | Power et al., 2012 |
| 194 | -10 | -2 | 42 | Cingulo-opercular | Power et al., 2012 |
| 195 | 37 | 1 | -4 | Cingulo-opercular | Power et al., 2012 |
| 196 | 13 | -1 | 70 | Cingulo-opercular | Power et al., 2012 |
| 197 | 7 | 8 | 51 | Cingulo-opercular | Power et al., 2012 |
| 198 | -45 | 0 | 9 | Cingulo-opercular | Power et al., 2012 |
| 199 | 49 | 8 | -1 | Cingulo-opercular | Power et al., 2012 |
| 200 | -34 | 3 | 4 | Cingulo-opercular | Power et al., 2012 |
| 201 | -51 | 8 | -2 | Cingulo-opercular | Power et al., 2012 |
| 202 | -5 | 18 | 34 | Cingulo-opercular | Power et al., 2012 |
| 203 | 36 | 10 | 1 | Cingulo-opercular | Power et al., 2012 |
| 204 | -7 | -52 | 61 | Sensory/somatomotor | Power et al., 2012 |
| 205 | -14 | -18 | 40 | Sensory/somatomotor | Power et al., 2012 |
| 206 | 0 | -15 | 47 | Sensory/somatomotor | Power et al., 2012 |
| 207 | 10 | -2 | 45 | Sensory/somatomotor | Power et al., 2012 |
| 208 | -7 | -21 | 65 | Sensory/somatomotor | Power et al., 2012 |
| 209 | -7 | -33 | 72 | Sensory/somatomotor | Power et al., 2012 |
| 210 | 13 | -33 | 75 | Sensory/somatomotor | Power et al., 2012 |
| 211 | -54 | -23 | 43 | Sensory/somatomotor | Power et al., 2012 |
| 212 | 29 | -17 | 71 | Sensory/somatomotor | Power et al., 2012 |
| 213 | 10 | -46 | 73 | Sensory/somatomotor | Power et al., 2012 |
| 214 | -23 | -30 | 72 | Sensory/somatomotor | Power et al., 2012 |
| 215 | -40 | -19 | 54 | Sensory/somatomotor | Power et al., 2012 |
| 216 | 29 | -39 | 59 | Sensory/somatomotor | Power et al., 2012 |
| 217 | 50 | -20 | 42 | Sensory/somatomotor | Power et al., 2012 |
| 218 | -38 | -27 | 69 | Sensory/somatomotor | Power et al., 2012 |
| 219 | 20 | -29 | 60 | Sensory/somatomotor | Power et al., 2012 |
| 220 | 44 | -8 | 57 | Sensory/somatomotor | Power et al., 2012 |
| 221 | -29 | -43 | 61 | Sensory/somatomotor | Power et al., 2012 |
| 222 | 10 | -17 | 74 | Sensory/somatomotor | Power et al., 2012 |
| 223 | 22 | -42 | 69 | Sensory/somatomotor | Power et al., 2012 |
| 224 | -45 | -32 | 47 | Sensory/somatomotor | Power et al., 2012 |
| 225 | -21 | -31 | 61 | Sensory/somatomotor | Power et al., 2012 |
| 226 | -13 | -17 | 75 | Sensory/somatomotor | Power et al., 2012 |
| 227 | 42 | -20 | 55 | Sensory/somatomotor | Power et al., 2012 |
| 228 | -38 | -15 | 69 | Sensory/somatomotor | Power et al., 2012 |
| 229 | -16 | -46 | 73 | Sensory/somatomotor | Power et al., 2012 |
| 230 | 2 | -28 | 60 | Sensory/somatomotor | Power et al., 2012 |
| 231 | 3 | -17 | 58 | Sensory/somatomotor | Power et al., 2012 |
| 232 | 38 | -17 | 45 | Sensory/somatomotor | Power et al., 2012 |
| 233 | -49 | -11 | 35 | Sensory/somatomotor | Power et al., 2012 |
| 234 | 36 | -9 | 14 | Sensory/somatomotor | Power et al., 2012 |
| 235 | 51 | -6 | 32 | Sensory/somatomotor | Power et al., 2012 |
| 236 | -53 | -10 | 24 | Sensory/somatomotor | Power et al., 2012 |
| 237 | 66 | -8 | 25 | Sensory/somatomotor | Power et al., 2012 |
| 238 | 47 | -30 | 49 | Sensory/somatomotor | Power et al., 2012 |
| 239 | -2 | -35 | 31 | Memory | Power et al., 2012 |
| 240 | -7 | -71 | 42 | Memory | Power et al., 2012 |
| 241 | 11 | -66 | 42 | Memory | Power et al., 2012 |
| 242 | 4 | -48 | 51 | Memory | Power et al., 2012 |
| 243 | 2 | -24 | 30 | Memory | Power et al., 2012 |
| 244 | 27 | -16 | -19 | Memory | Yarkoni et al., 2011 |
| 245 | -27 | -20 | -18 | Memory | Yarkoni et al., 2012 |

Abbreviations: ROI, region of interest; MNI, Montreal Neurological Institute.
